# Diagnosis and Treatment of Acute Periprosthetic Infections with the BioFire^®^ System within a Time-Dependent and Bacterium-Dependent Protocol: Review and Prosthesis-Saving Protocol

**DOI:** 10.3390/biomedicines12092082

**Published:** 2024-09-12

**Authors:** Rudy Sangaletti, Luca Andriollo, Alice Montagna, Simone Franzoni, Paolo Colombini, Loris Perticarini, Francesco Benazzo, Stefano Marco Paolo Rossi

**Affiliations:** 1 Sezione di Chirurgia Protesica ad Indirizzo Robotico—Unità di Traumatologia dello Sport, Ortopedia e Traumatologia, Fondazione Poliambulanza, 25124 Brescia, Italy; 2 Ortopedia e Traumatologia, Università Cattolica del Sacro Cuore, 00168 Rome, Italy; 3 Ortopedia e Traumatologia, Università degli Studi di Pavia, 27100 Pavia, Italy; 4 Biomedical Sciences Area, IUSS Istituto Universitario di Studi Superiori, 27100 Pavia, Italy

**Keywords:** acute periprosthetic infections, BioFire system, bacterium-dependent, DAPRI, DAIR, PJI

## Abstract

Despite ongoing efforts to enhance diagnostic and treatment processes, the success rate for eradicating infections, particularly prosthetic joint infections (PJIs), currently stands at around 50%. For acute infections occurring shortly after arthroplasty, guidelines recommend a treatment known as DAIR (debridement, antibiotics, and implant retention). This approach is suggested for infections within 30 days post-arthroplasty or with less than 3 weeks of symptoms, provided that there is a stable implant and adequate soft-tissue mass. Several authors have suggested extending the use of DAIR beyond the initial 3-week period in specific cases. This extension practice seems increasingly feasible due to the rapid diagnostic capabilities offered by BioFire^®^. This technology allows for quick pathogen identification, aiding in the exclusion of cases that do not fit the criteria for the DAIR/DAPRI (debridement, antibiotic pearls and retention of the implant) protocol based on pathogen identification. The aim of this review is to re-examine the current literature on acute infections and present our proposed “prosthesis-saving” protocol, which integrates the BioFire^®^ molecular diagnostic system. Continued research and assessment of the efficacy and safety of these protocols, especially regarding extended treatment timelines, are crucial for advancing the management of acute infections and enhancing outcomes for PJI patients.

## 1. Introduction

The periprosthetic joint infection (PJI) is the most common and devastating complication of joint replacement, with an average rate of approximately 1–2% [1,2]. PJI may require multiple hospitalizations and invasive treatments with high risk of significant adverse events and medicolegal issues. As a consequence, the overall cost of PJI treatment is among the highest among orthopedic procedures [3,4,5,6].

PJI is a type of infection caused by biofilm formation on the surface of an inert implant. PJI is a complex entity that differs from bone and joint infections in that the colonized implant becomes a persistent reservoir of microorganisms, increasing the difficulty of successfully diagnosing and treating the infection [7].

Microbiological analysis is currently the most reliable tool for guiding PJI treatment. Since the early days of implant arthroplasty in orthopedics, the etiological diagnosis, which is based on microbiological examination, leads the specific antibiotic treatment.

Staphylococci, specifically *S. aureus* and *Staphylococcus epidermidis*, are the most common biofilm-forming bacteria. Along with *Pseudomonas aeruginosa*, these organisms constitute nearly 75% of the biofilms found on medical devices [8,9].

Microorganism identification plays an important role in confirming infection, and various diagnosis guidelines have been discussed and proposed by various organizations (Infectious Diseases Society of America—IDSA; Musculoskeletal Infection Society—MSIS; International Consensus Meeting of Philadelphia—ICM; and European Bone and Joint Infection Society—EBJIS) [2,10,11]. The identification of the same microorganisms isolated from two or more cultures or the presence of a sinus tract communicating with the joint are among the widely accepted major criteria for PJI, though some differences are found in the minor criteria, which are constantly revised and improved.

Traditional culture techniques have limitations in identifying microorganisms in prosthetic infection, even when repeated twice for confirmation. According to a recent systematic review, false negatives (negative cultures) range from 5% to 42% of PJI, due to sampling techniques, prior antibiotics, insufficient culture time, chronic infections, and, perhaps, other unknown causes, compromising the diagnosis and treatment of PJI [12].

The risk of PJI is a critical consideration within joint-replacement procedures. Understanding the various risk factors is pivotal in preventing, diagnosing, and effectively treating infections. Risk factors for the onset of PJI encompass a range of preoperative, intraoperative, and postoperative elements. Preoperative factors include pre-existing medical conditions such as diabetes, obesity, and immunosuppression, as well as the overall health status of the patient. Adherence to infection prevention protocols, hygiene, and dental health can also impact the risk of PJI. During surgery, factors such as the duration of the procedure, surgical techniques employed, environmental hygiene, and the management of prostheses and surgical instruments can influence the risk of infection. Proper sterilization of surgical tools and adherence to aseptic practices are crucial in reducing the risk of contamination. Post-surgery, infections may arise due to inadequate wound management, the presence of drains or medical devices, or insufficient hygiene in wound care. Postoperative management, including antibiotic therapy and preventive measures against nosocomial infections, can significantly influence the risk of PJI. Patient age, type of surgical intervention, pre-existing medical conditions, and adherence to infection prevention protocols are all factors that can impact the risk of PJI [2,13].

Recognizing and mitigating these risk factors is crucial in reducing the incidence of PJI. Surgical guidelines, stringent hygiene practices, adherence to sterilization protocols, and appropriate use of prophylactic antibiotics are all critical components in reducing the risk of periprosthetic joint infection and improving the overall outcomes of joint-replacement procedures.

Despite the constant effort to improve the processes of diagnosis and treatment, the success of the eradication of infections is now around 50% [14]. Furthermore, PJI treatment is related to poor functional outcomes and even to depressed and discouraged patients [15]. Therefore, orthopedic surgeons are often tempted to deny the problem by delaying the diagnosis and treatment [16]. However, the delay in diagnosis and treatment turns acute infections into even more serious pathologies, as an early diagnosis and a consequent targeted treatment can save the implant, with a great clinical impact for the patient and an economic impact for the healthcare system. The aim of this discursive review is to re-evaluate the literature available today on acute infections and to present our “prosthesis-saving” protocol integrating the BioFire^®^ Joint Infection Panel (BioFire Diagnostics, LLC, Salt Lake City, UT, USA).

## 2. Impact of BioFire^®^ on the Approach to Periprosthetic Joint Infection

As a consequence of the importance of the PJI diagnosis, an increased effort has been made to state a definition. Acute PJIs are primarily attributed to intraoperative contamination, potentially resulting in early or delayed infections based on pathogen virulence and the patient’s response [17]. Conversely, hematogenous seeding, a less frequent occurrence, becomes suspected when an infection emerges years after the initial arthroplasty [2,13].

Both intraoperative and hematogenous infections typically display an acute onset, despite differing pathogeneses, and are classified as ‘acute infections’ based on symptom duration. Consequently, treatment approaches often overlap despite their distinct origins [18]. Different classification systems are used to distinguish acute, late chronic, and late acute PJI. Hence, the definition of acute infection is extremely important and is related to the probability that the infection is supported by a stable biofilm that cannot be eradicated with local therapy and systemic antibiotics. [18] Tsukayama et al. suggested a system that divides the onset of infection into four groups: positive intraoperative cultures, early postoperative infection (<4 weeks), late chronic infection (>4 weeks, indolent onset), and acute hematogenous (acute postoperative onset with symptoms for less than 4 weeks) [13]. Contrariwise, the definition proposed by Zimmerli et al. is very different, as it divides PJIs in early (occurring within 3 months postoperatively), delayed (3–24 months) and late (>24 months) [1]. The variability in the time cutoffs used to define acute infection is related to the ability of the specific pathogen to create a biofilm [1,17].

The most widely accepted therapeutic indication in cases of acute infection before the formation of the biofilm is DAIR (debridement, antibiotics, and implant retention). DAIR consists of sampling periprosthetic tissue, removing all infected and necrotic tissue, replacing all modular implants, and plentiful lavage [19]. The literature shows a high variability in the efficacy rate of DAIR. In most studies evaluating small, selected groups the efficacy levels presented lie between 60 and 80%, while in cohorts with more than 100 patients (including both hip and knee PJI), success rates decrease to 31–78%. In order to improve the efficacy of DAIR, various treatment protocols have been proposed, which include physical and chemical techniques for the eradication of biofilm or local antibiotic carriers [19].

For acute infections with a stable implant and adequate soft-tissue mass, the latest guidelines recommend implant retention treatment (also referred to as DAIR) for PJI occurring within 30 days after arthroplasty, or with less than 3 weeks of symptoms [2].

The variability in the efficacy of DAIR is related to the time of the surgical intervention. A review published by Horriar et al. evaluated the efficacy of DAIR in acute and chronic PJIs and concluded that the earlier the surgical intervention is performed, the higher is the success rate of the procedure [20]. As a consequence, the therapeutic indication for DAIR is often limited to early or acute hematogenous infections. Even though there is no evidence that, in appropriately selected cases, DAIR should not be considered outside strict time limits, it is important to point out that after four to six weeks, there is a higher probability of mature biofilm and consequently a more difficult eradication required in order to remove all infected material [21,22]. Studies have reported some success with DAIR treatment in chronic situations, but success rates are known to be lower in this situation [19,23].

One key factor for the success of the DAIR or DAPRI (debridement antibiotic pearls and retention of the implant) procedure is biofilm formation and its crucial role in the pathogenesis and course of the PJI. The formation of the biofilm depends on the time passed since the onset of the infection and on the type of bacterium. As a consequence, within 3 months, the element that most modifies the prognosis of the treatment is the type of pathogen and our ability to eradicate the biofilm. Difficult-to-treat (DTT) bacteria are defined as microorganisms for which a highly bioavailable biofilm-active antibiotic is not available, and their treatment is even more challenging [24]. Consequently, biofilm-active antibiotic therapies include fluoroquinolones in PJI in case of Gram-negative biofilm-forming pathogens and rifampicin in PJI caused by staphylococci or other Gram-positive biofilm-forming pathogens [1,24,25]. The literature consistently supports the view that antimicrobial treatments targeting biofilms are more effective than antibiotics without biofilm-specific activity. This efficacy is evidenced by improved outcomes in infection management, decreased pain intensity, and enhanced joint functionality [26,27]. Several studies concluded that when the pathogen causing PJI is *Staphylococcus aureus*, it represents an independent risk factor for treatment failure [28,29,30,31]. Even-lower success rates associated with DAIR are reported in the case of MRSA [32,33]. Furthermore, in cases of Gram-negative microorganisms, the outcomes are shown to be more variable as compared to Gram-positive organisms [34,35]. Also, fungal or multi-drug resistant organisms represent a risk factor for a decreased success rate [36].

As far as contraindications to performing a DAIR procedure, a persistent sinus is considered a risk factor for failure [37,38,39]. Moreover, immunosuppressed patients are not suitable for DAIR, as a competent immune system is required to eradicate the infection. [37].

In contrast, DAIR should be considered in cases of patients presenting significant morbidity who are not eligible for more-demanding surgery [38,40,41].

The evidence presented suggests that the effectiveness of DAIR procedures relies heavily on prompt intervention and the precision of treatment, especially in targeting the particular pathogen causing the infection. Hence, immediate identification of the causative agent becomes crucial. However, certain slow-growing bacteria, like *Cutibacterium acnes* (*C. acnes*), might demand an incubation period of up to two weeks in order to confirm their role in the infectious process [10]. This circumstance leads to prolonged durations, both in the identification process of the infection and the commencement of therapeutic measures, subsequently resulting in an escalation of associated complications. Additionally, the precise determination of the bacterial entities might encounter obstacles due to contamination stemming from commensal bacteria and the possibility of cross-contamination during the phases of storage and incubation. To mitigate the time frame necessary for the growth of microorganisms in culturing, patients are administered preventive treatments comprising broad-spectrum antibiotics, a practice that poses challenges due to the escalating prevalence of antibiotic-resistant strains among pathogens. Consequently, there remains an absence of specific knowledge pertaining to the identification of the causative pathogen and its antibiotic resistance profile prior to the initiation of therapeutic interventions, potentially leading to the application of antibiotic agents ordinarily reserved for the treatment of pathogen strains which are more aggressive. The FDA has recently approved the rapid molecular BioFire^®^ Joint Infection Panel (BJIP) for synovial fluid [42].

The BioFire^®^ System utilizes cutting-edge multiplex PCR technology to conduct syndromic tests, enabling the simultaneous detection of various pathogens associated with specific clinical syndromes.

The BioFire^®^ FilmArray System conducts multiplex PCR syndromic tests. This method involves the amplification of multiple DNA targets in a single polymerase chain-reaction, thereby streamlining the diagnostic process [42].

The preparation and insertion of a panel pouch into a BioFire^®^ System instrument requires only two minutes of hands-on time. Subsequently, the entire testing process, including sample integration and automated results analysis, unfolds within the pouch. The system’s key innovation lies in its array design, which features individual wells arranged on a single plate. This configuration enables multiplex PCR reactions to occur concurrently without the risk of cross-contamination [42].

Each well in the array contains a distinct set of primers designed to detect specific targets listed on the panel menu. Following the completion of PCR cycles, the presence or absence of targeted organism DNA is determined based on the wells’ results.

BJIP is designed to detect 15 Gram-positive (seven anaerobes) bacteria, 14 Gram-negative bacteria (one anaerobe), two yeasts, and eight antimicrobial resistance (AMR) genes from synovial fluid specimens in an hour, allowing for rapid microbiological identification and subsequently targeted adequate antimicrobial therapy. Appendix A lists the pathogens included in the panel. Using synovial fluid culture as a reference standard results in a sensitivity of 90.6% and specificity of 99.8% [42].

Drawing from the existing literature, numerous authors have put forward the idea of expanding the time frame for implementing the DAIR approach beyond the usual 3-week period in certain specific scenarios. The use of BioFire^®^, a swift diagnostic tool, could play a pivotal role in promptly identifying the causative pathogens and effectively excluding cases involving DTT pathogens from the standard DAIR treatment protocol. This could potentially lead to an extension of the use of DAIR beyond three weeks, primarily due to its rapid diagnostic capabilities. The primary objective of this manuscript is to introduce and present our proposed extension protocol for the DAPRI approach. The goal of this study is to share this protocol while anticipating scientific endorsement for its broader systematic application.

## 3. Proposal for a Prosthesis-Saving Protocol

The protocol for managing PJI has undergone significant transformation thanks to the implementation of BioFire^®^ technology. For all instances of infection lasting less than three months, a comprehensive set of examinations is conducted. This includes blood tests encompassing a complete blood count, ESR, and PCR, alongside specific X-ray imaging for the affected joint, as well as ultrasound-guided arthrocentesis. Synovial fluid analysis involves a standard culture, chemical–physical assessment for leukocyte count and polymorphonuclear percentage, and the utilization of BioFire^®^ technology. This technology detects a wide spectrum of 31 microorganisms and 8 antimicrobial resistance genes, providing results within a remarkably swift timeframe of just 1 h [38].

In the context of temporal considerations, we have devised and delineated two separate and distinct protocols for addressing periprosthetic infections.

**Figure 1:**
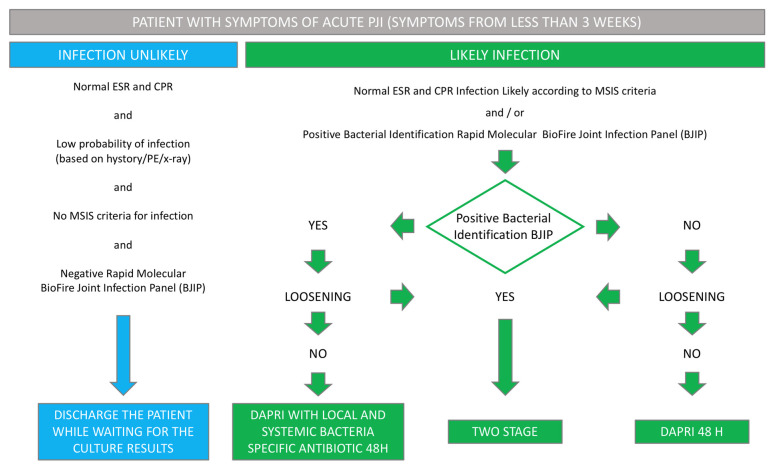
Protocol A: algorithm for patients with symptoms of acute PJI (for less than 3 weeks) [PJI: Periprosthetic Joint Infection; ESR: Erythrocyte Sedimentation Rate; CPR: C-Reactive Protein; MSIS: Musculoskeletal Infection Society; PE: Physical Examination; DTT: Difficult-to-Treat].

Protocol A (Figure 1) involves the following: As per the existing body of literature, patients presenting symptoms for a duration of less than three weeks showcase a higher probability of successfully resolving infections and preserving the implant, regardless of the causative pathogen. This subset of patients undergoes prompt initiation of the DAPRI protocol within a 24 h timeframe post-diagnosis, unless indications of implant instability are evident. The utilization of BioFire^®^ technology facilitates immediate identification and susceptibility testing of the pathogen. Consequently, the tailored DAPRI treatment is meticulously designed to combat the specific bacterium identified, incorporating systemic and locally targeted antibiotics known to be effective against the identified pathogen.

**Figure 2:**
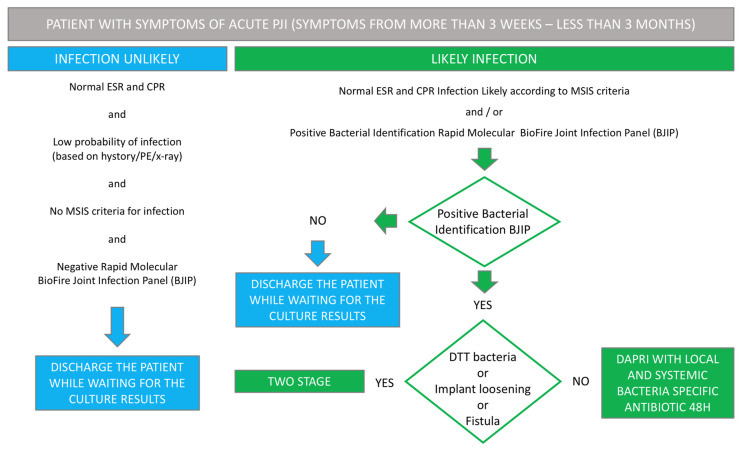
Algorithm for patients with symptoms of acute PJI (for more than 3 weeks–less than 3 months) [PJI: Periprosthetic Joint Infection; ESR: Erythrocyte Sedimentation Rate; CPR: C-Reactive Protein; MSIS: Musculoskeletal Infection Society; PE: Physical Examination; DTT: Difficult-to-Treat].

Conversely, in Protocol B (Figure 2), in cases where symptoms persist for a duration extending beyond three weeks yet resolve within three months, the implementation of the DAPRI protocol is considered only if other predisposing factors for treatment failure, such as the presence of DTT bacteria or the existence of a fistula, are absent. It is noteworthy that the initiation of the DAPRI protocol is contraindicated in scenarios in which implant instability is evident or in situations where achieving adequate skin coverage is deemed unattainable.

The DAPRI surgical procedure involves three steps: (1) identification of the biofilm, (2) removal of the biofilm, and (3) prevention of PJI recurrence.

Before the surgical approach, the intra-articular injection of 50 cc of diluted methylene blue (40 mL saline and 10 mL of 0.5% methylene blue solution) is performed so that the bacterial biofilm is highlighted, in order to simplify the identification of the biofilm after capsulotomy. Afterwards, flexion and extension of the joint is performed multiple times to distribute the methylene blue inside the joint and the excessive color stain is removed via arthrocentesis. After the surgical approach and capsulotomy, blue staining of all intra-articular surfaces was identified, allowing for a “tumor-like” synovectomy and removal of all the biofilm from the intra-articular lining near the intra-articular infection. After this eradication, the biofilm is treated with mechanical and chemical means [43]. Mechanical removal is based on a 2% chlorhexidine gluconate-added brush used for scrubbing on all visible implant components, as described by Indelli et al. [43].

Since 2019, the authors of this study have also used an acetic acid and benzalkonium chloride (BZK)-based surgical lavage solution (Bactisure, Zimmer–Biomet, Warsaw, IN, USA) as an antimicrobial solution. The application was always followed by extensive pulse irrigation with 9 liters (L) of saline containing povidone iodine to reduce the local toxicity of acetic acid [44,45]. When the intra-articular space was considered to be free of biofilm, the wound was temporarily closed, all the surgeons left the operating room, and the surgical drapes and contaminated instruments were removed from the surgical field. The surgical field was then prepared with sterile instruments, the surgical team scrubbed again, and then re-entered the operating room after changing their surgical gowns. At this point, a new instruments table is prepared in a standard fashion. As soon as these preparation phases are completed, the wound is then re-opened, further irrigation of the joint is performed using a saline pulse, and the new modular components are implanted. Furthermore, 10 mL of calcium sulphate antibiotic-added beads (Stimulan, Biocomposites, Keele, UK) are prepared on the back table and placed in the joint before closure. The antibiotic added to the beads is always selected according to the antibiogram or the preoperative molecular testing result obtained at the time of microorganism identification. After the placement of an intra-articular Hemovac, the soft tissues are closed. Post-operative antibiotic treatment lasts for a minimum of 12 weeks, as recommended by the infectious disease specialists; a six-week course of intravenous antibiotic therapy is then followed by a six-week course of oral antibiotic therapy.

## 4. State-of-Art and Future Perspectives

Diagnostic strategies for periprosthetic joint infections have expanded to include a diverse range of tools and techniques. Alongside conventional microbiological investigations like bacterial cultures and antibiotic sensitivity tests, advanced imaging methods such as magnetic resonance imaging (MRI) and computed tomography (CT) play pivotal roles in providing crucial diagnostic insights. Bacterial cultures and sensitivity tests remain the primary means for identifying specific infecting microorganisms and determining their susceptibility to antibiotics. Serological tests measuring inflammatory markers like C-reactive protein (CRP) and erythrocyte sedimentation rate (ESR) complement the diagnostic process by indicating elevated levels in the presence of an infection. Advanced imaging techniques such as MRI and CT offer detailed insights. MRI, providing high-resolution imaging without radiation exposure, assesses soft-tissue changes, detects fluid collections, and identifies bone-related issues around the joint prosthesis. CT scans provide detailed images of bone structures, aiding in the identification of defects, fractures, and infection-related complications. Nuclear medicine scans like bone scintigraphy, indium-labeled leukocyte scintigraphy, and fluorodeoxyglucose–positron emission tomography (FDG-PET) can detect increased metabolic activity or leukocyte accumulation, highlighting potential infection sites.

Ultrasound, though less common, assists in assessing soft-tissue changes, detecting fluid collection points, and guiding joint aspirations for diagnostics.

Histological analysis of biopsied periprosthetic tissues under a microscope helps identify infection signs like inflammatory cells or bacterial biofilms. Clinical evaluation, considering patient history, symptoms, and physical-examination findings, forms an integral part of the diagnostic process. Combining these diagnostic modalities in a multidisciplinary approach involving orthopedic surgeons, infectious disease specialists, radiologists, and microbiologists enhances the accuracy of the diagnosis of periprosthetic joint infections. It aids in formulating appropriate treatment strategies, including antibiotic selection and the potential need for surgical intervention [41,42].

However, it is important to note that these investigations may present limitations in distinguishing between infection and non-infectious inflammatory responses, often requiring further invasive evaluations or more specific diagnostic tests [41,42].

The introduction of serum biomarkers such as C-reactive protein (CRP) and lactate dehydrogenase (LDH) has enhanced the diagnosis of periprosthetic joint infections. While these biomarkers are non-specific to infection, they can provide critical indications of inflammatory activity and infection presence, contributing to a more comprehensive diagnostic framework [41,42].

Furthermore, the utilization of molecular diagnostic techniques like polymerase chain reaction (PCR) and DNA sequencing is emerging as a promising frontier in the diagnosis of periprosthetic joint infections. These techniques enable the detection of specific pathogens, improving diagnostic sensitivity, and reducing wait times, compared to traditional cultures [33,43].

However, despite the advantages offered by molecular techniques, diagnosing periprosthetic joint infections remains complex. The presence of bacterial biofilms can make pathogen detection challenging, and the interpretation of molecular results might be influenced by prior antibiotic exposure or variability in sample preparation [33,43].

The purpose of this article is to present our “prosthesis-saving” protocol with the inclusion of molecular diagnosis. Our research objective aims to enhance the diagnosis and treatment of acute infections in a manner that swiftly and specifically combats bacteria before the formation of biofilm, eliminating the necessity for a two-stage treatment. Multiple studies in the literature have established that septic revision surgery incurs exceptionally high costs, which further escalates in cases where the procedure proves unsuccessful. Yao et al. previously discussed the elevated costs linked with TKA (total knee arthroplasty) performed for PJI compared to those undertaken for aseptic revision ($56,900 versus $24,630, *p* < 0.001) [46,47]. Thus, we firmly believe that the utilization of BioFire^®^ has a substantial impact on both costs and clinical outcomes.

A prompt and precise diagnosis of PJI is crucial for timely medical and surgical interventions, mitigating the risk of disability and preserving the implant in cases of acute infections. In 2011, the Musculoskeletal Infection Society (MSIS) and the Infectious Diseases Society proposed criteria to define PJI, aiming to establish a gold standard definition that would enhance diagnostic progress [44]. These criteria were further refined and integrated with modern diagnostic tests by the Consensus Meeting in Philadelphia in 2018 [10]. The updated criteria introduced more-recent diagnostic tests, supported by 97.7% sensitivity and 99.5% specificity, compared to the 86.9% sensitivity and 79.3% specificity of the 2011 MSIS definition [48]. Nevertheless, the recent literature has revealed low sensitivity for MSIS criteria, with a few studies reporting rates below 50%, while none have specifically described the sensitivity of MSIS criteria in acute infections versus chronic ones [49].

Additionally, recent research underscores the importance of pathogen identification when selecting a treatment option [24]. Swift detection of resistant strains such as methicillin-resistant *S. aureus* (MRSA), extended-spectrum beta-lactamase producers (ESBL), and vancomycin-resistant enterococci (VRE) by the BioFire^®^ Joint Infection Panel (BJIP) significantly contributes to the use of the appropriate antimicrobial therapy and infection control precautions to prevent further transmission.

Numerous studies have assessed the ability of molecular methods to improve the speed and accuracy of pathogen diagnosis in PJI. Compared to the traditional culture, 16S rRNA PCR and next-generation sequencing exhibit superior sensitivity [43,50].

Nevertheless, despite their effectiveness, these tests often come at a high cost and may not hasten the process of diagnosis and subsequent treatment initiation, which is crucial for improving the effectiveness of treating acute infections. In contrast, the BioFire^®^ panel designed for acute infections provides swift identification of the pathogens included within its targeted study panel. This accelerated identification facilitates the prompt initiation of specific bacterial treatment for acute infections. Moreover, notably, it extends the treatment window beyond the typical 3 weeks, sometimes up to 3 months, particularly in selected cases where there is a high likelihood of efficacy. This extension aids in ruling out DTT bacteria for DAIR (debridement, antibiotics, and implant exchange) treatment strategies in a few cases. The BioFire^®^ panel’s molecular diagnostic system’s accuracy in vitro reports a sensitivity and specificity of 90% and 100%, respectively, illustrating its superiority over standard cultures [51].

Pascual et al. conducted a study at 34 clinical sites across 19 countries in Europe and the Middle East from March 2021 to June 2022, evaluating the effectiveness of the BJIP [52]. The study collected 1527 samples from patients suspected of having septic arthritis or PJI, achieving an agreement rate of 88.4% and 85% with synovial fluid cultures, respectively. The BJIP identified a greater number of positive samples and a wider range of microorganisms, compared to synovial fluid cultures, especially noting higher detection rates for *Staphylococcus aureus*, *various Streptococcus species*, *Enterococcus faecalis*, *Kingella kingae*, *Neisseria gonorrhoeae*, and anaerobic bacteria. The findings indicate that the BJIP could significantly enhance patient management and the effectiveness of antimicrobial treatments, underscoring its vital role in medical practice.

In a study by Gardete-Hartmann et al. involving 268 synovial fluid samples from 195 patients undergoing acute or chronic revision of total hip or knee arthroplasty, an evaluation using the BJIP on synovial fluid was carried out and the findings were juxtaposed with those from traditional cultures [53]. It was found that 19 out of 195 cases (9.7%) could have benefited from more precise management if assessed with the BJIP. The authors concluded that despite certain microbial-detection limitations, the BJIP proved clinically valuable in its ability to enhance traditional culture-based methods, especially in cases in which the PJI microbiological results were ambiguous.

Schoenmakers et al. highlighted the distinct clinical benefits of the BJIP for patients suspected of having a late acute (hematogenous) PJI, noting its clear advantages in these cases [54]. However, they observed a lower clinical benefit for patients with an early acute (post-operative) PJI. This reduced efficacy is attributed to the exclusion of certain relevant microorganisms, such as *Staphylococcus epidermidis*, from the panel.

However, Hoffman et al. evaluated 57 PJIs and reported an overall sensitivity of 56% and specificity of 100%, obtaining the same diagnostic sensitivity as conventional cultures [55]. The authors concluded that most infected cases with negative BioFire^®^ results were also negative in traditional cultures or caused by pathogens not included in the BioFire^®^ repertoire.

In the context of chronic infections, the potential of BioFire^®^ for diagnosis might face limitations due to the reduced impact of a swift diagnosis on the treatment decision and the involvement of organisms displaying lower virulence. Nevertheless, an essential advantage of BioFire^®^, encouraging its application even in chronic situations, lies in its capacity to identify pathogens effectively. This proves particularly advantageous when considering patients who have undergone previous antibiotic therapy, as BioFire^®^ demonstrates a greater probability of identifying the specific causative agent, compared to traditional culture methods. This aspect becomes notably critical in chronic scenarios in which traditional culture techniques might encounter challenges in detecting pathogens due to prior exposure to antibiotics.

Despite finding support in the existing literature, further studies are necessary to validate our proposed protocol. Specifically, it is imperative to conduct a multicenter study to initially compare the sensitivity of MSIS criteria with and without the integration of BioFire^®^ in acute infections. Subsequent research should focus on assessing whether the recommended diagnostic and treatment protocol yields higher success rates and analyzing the financial implications of adopting the BioFire^®^ System as essential objectives.

In conclusion, the recent literature states that molecular diagnostic technologies provide a cutting-edge approach for the rapid identification of microorganisms when a joint infection is clinically suspected [56].

## Data Availability

Not applicable.

## Appendix A

The BioFire^®^ Joint Infection Panel includes a wide range of pathogens such as Gram-negative bacteria like *Bacteroides fragilis*, *Citrobacter*, *Enterobacter cloacae* complex, *Escherichia coli*, *Haemophilus influenzae*, *Kingella kingae*, *Klebsiella aerogenes*, *Klebsiella pneumoniae* group, *Morganella morganii*, *Neisseria gonorrhoeae*, *Proteus* spp., *Pseudomonas aeruginosa*, *Salmonella* spp., and *Serratia marcescens*. It also tests for yeast, specifically, *Candida* spp., including *Candida albicans*. The panel also identifies Gram-positive bacteria such as *Anaerococcus prevotii*/*vaginalis*, *Clostridium perfringens*, *Cutibacterium avidum*/*granulosum*, *Enterococcus faecalis*, *Enterococcus faecium*, *Finegoldia magna*, *Parvimonas micra*, *Peptoniphilus*, *Peptostreptococcus anaerobius*, *Staphylococcus aureus*, *Staphylococcus lugdunensis*, and various Streptococcus species, including *Streptococcus agalactiae*, *Streptococcus pneumoniae*, and *Streptococcus pyogenes*.

The BioFire^®^ Joint Infection Panel also includes the means of detection of the key antimicrobial resistance genes which are critical for guiding appropriate treatment strategies. The panel identifies several carbapenemases, such as IMP, KPC, NDM, OXA-48-like, and VIM, which are important markers of resistance to carbapenem antibiotics. Additionally, it detects extended-spectrum beta-lactamases (ESBL), specifically, CTX-M, which confer resistance to a broad range of beta-lactam antibiotics. The panel tests for methicillin resistance through the detection of mecA/C genes and the mec right extremity junction (MREJ), which are associated with methicillin-resistant Staphylococcus aureus (MRSA). Furthermore, it includes markers for vancomycin resistance, specifically the vanA and vanB genes, which are critical for identifying strains resistant to this last-resort antibiotic.
